# Nomograms for predicting the prognostic value of serological tumor biomarkers in colorectal cancer patients after radical resection

**DOI:** 10.1038/srep46345

**Published:** 2017-04-18

**Authors:** Qingguo Li, Weixing Dai, Yaqi Li, Ye Xu, Xinxiang Li, Sanjun Cai

**Affiliations:** 1 Department of Colorectal Surgery, Fudan University Shanghai Cancer Center, Shanghai 200032, China; 2 Department of Oncology, Shanghai Medical College, Fudan University, Shanghai 200032, China

## Abstract

A wide range of serum tumor biomarkers, including CA19-9, CA242, CA72-4, CA50, and CA125, has been studied in association with colorectal cancer (CRC). However, few previous studies have comprehensively considered the above tumor biomarkers to assess their clinical significance in predicting prognosis. Data from Fudan University Shanghai Cancer Center (FUSCC) between January 1, 2007 and December 30, 2012 was retrospectively analyzed. Univariate and multivariate analyses were performed to assess the association between prognostic factors and survival outcomes. Nomograms were established based on multivariate Cox regression model analysis for overall survival (OS) and disease free survival (DFS), and c-indexes were 0.772 (95% CI: 0.724-0.820) and 0.715 (95% CI: 0.678–0.752), respectively. Subgroup analyses according to CEA status (high/normal) suggested that CA724 was the only independent prognostic factor for OS (*P* = 0.001) and DFS (*P* < 0.001) in the CEA-high group, while, in the CEA-normal group, the only independent prognostic factor for OS (*P* = 0.031) and DFS (*P* = 0.043) was CA50. CA50 and CA724 could supplement CEA in monitoring recurrence and metastasis. Accordingly, nomograms based on CEA, CA50, CA724 and other clinical-pathological factors could improve prognosis prediction for colorectal cancer patients.

Colorectal cancer (CRC) is one of the most prevalent types of cancer, ranking as the third most common malignancy and the fourth leading cause of cancer deaths worldwide^1^. Radical resection is the only potential cure. However, occult metastasis often diminishes its therapeutic effectiveness, with over 45% of patients dying from recurrence despite adjuvant therapy^2^. Early detection of recurrence and metastasis is critical in reducing mortality rates. Although triple-phase helical computed tomography (CT) and positron emission tomography (PET) may help to detect metastasis after radical colectomy, they cannot repeatedly be performed, and the cost is very high. Some oncogenes or tumor suppressors were also proposed as predictors. However, these biomarkers were only validated in a small sample of patients, and some of them may be difficult to measure due to sophisticated and expensive laboratory techniques. There is an urgent need to identify a simple but cost-effective indicator for predicting the prognosis of patients. Serologic biomarkers, particularly those that can be monitored easily in a relatively noninvasive and cost-effective manner, would be helpful for choosing treatment strategies^3^.

Seven serological tumor biomarkers (CEA, CA19-9, CA242, CA72-4, CA50, CA125, and AFP) are routinely adopted in clinical practice to assist diagnoses and determine prognosis in gastroenterological cancers at our institute. The most common and best-studied biomarker for CRC is CEA, which can be used for early diagnosis, recurrence detection and prediction of survival outcomes^4^,^5^. However, CEA cannot specifically reflect the metastatic potential of the tumor, and CEA levels do not rise in some patients when recurrences or metastases occur. Thus, detection of other cancer biomarkers that supplement CEA may play important clinical roles in the early diagnosis of recurrences and metastases. A variety of serum tumor biomarkers has been studied in CRC, including CA19-9, CA242, CA72-4, CA50 and CA125. For example, the combination of CEA and CA242 achieved significantly higher sensitivity compared with the use of either biomarker for CRC^6^. Wang *et al*. demonstrated that the combined analysis of CEA, CA19-9, and CA242 could improve the accuracy of prognostic prediction in surgically treated CRC patients^7^. However, to our knowledge, there is no study in the literature that has comprehensively considered the above tumor biomarkers to evaluate their prognostic value.

The development of nomograms has led to their successful application in oncology prognostics in many studies. However, nomograms for predicting survival outcomes of CRC with serological tumor markers are scarce. In the present study, we evaluated the association between preoperative levels of tumor biomarkers and other baseline characteristics and developed nomograms to determine the value of tumor biomarkers for predicting the 5-year survival of patients with CRC who underwent radical resection.

## Results

### Patient Baseline Characteristics

The descriptive characteristics of eligible patients are displayed in Table 1. Of the 807 patients, 324 patients had tumors located in the colon, and 483 patients had tumors located in the rectum. Post-operative pathological tumor staging suggested that 64 patients were in the T1 stage, 146 patients in the T2 stage, 392 in the T3 stage, and 205 were in the T4 stage, while 468 patients were in the N0 stage, 202 in the N1 stage, and 137 in the N2 stage. Well to moderately differentiated tumors were observed in 575 patients, with poorly-differentiated tumors in 191 patients and unknown differentiation in the remaining 41 patients. The positive serum rates of CEA, CA125, CA19-9, CA50, CA242 and CA72-4 before surgery were 38.8% (313/807), 6.3% (51/807), 23.9% (193/807), 8.1% (65/807), 26.6% (215/807) and 18.3% (148/807), respectively.

### Prognostic Value of Tumor Biomarkers

**Figure 1:**
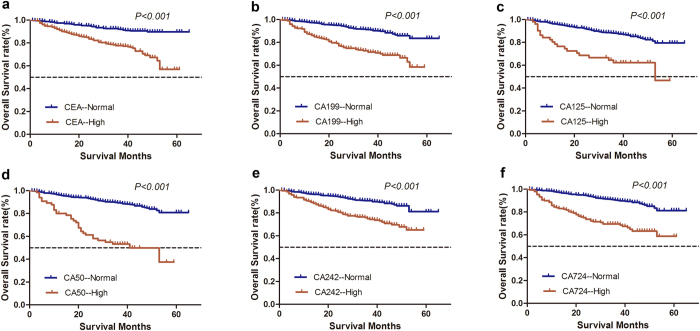
Comparison of overall survival according to six serological tumor biomarkers in patients with colorectal cancer after radical resection. A significant difference in the survival of patients was observed between tumor markers-high and tumor markers-normal subgroups (*P* < *0.05*).

The median follow-up time was 38.2 months. The 5-year OS was 75.0%, and the 5-year DFS was 61.0%. In univariate analysis, tumor length, T stage, N stage, tumor differentiation, venous invasion, perineural invasion, CEA, CA125, CA19-9, CA50, CA242, and CA724 showed significant association with OS (*P* < *0.05*; Fig. 1, Table 2). Factors that showed prognostic significance in univariate analysis were included in the multivariate analysis. Multivariate Cox regression analysis demonstrated that only T stage (*P* < *0.001*), N stage (*P* < *0.001*), CEA (*P* = 0.034), CA50 (*P* = 0.010), and CA724 (*P* < *0.001*) were independent prognostic factors for OS (Table 2).

**Figure 2:**
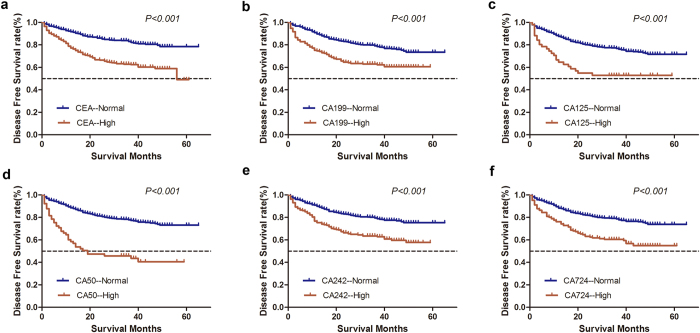
Kaplan-Meier survival curves depicting disease-free survival according to the level of preoperative tumor biomarkers. High preoperative tumor biomarkers indicate a shorter disease-free survival after radical resection in patients with colorectal cancer (*P* < *0.05*).

For univariate analysis of DFS, tumor length, tumor location, T stage, N stage, tumor differentiation, perineural invasion, venous invasion, CEA, CA125, CA19-9, CA50, CA242, and CA724 showed significant associations with DFS (*P* < *0.05*; Fig. 2, Table 2). In multivariate Cox regression analysis, T stage (*P* < *0.001*), N stage (*P* < *0.001*), CEA (*P* = 0.017), CA50 (*P* = 0.020), and CA724 (*P* = *0.001*) were independent prognostic factors for DFS (Table 2).

The different levels of CEA, CA50, and CA724 among patients with CRC in different groups are presented in Table 1. Those patients with high levels of serum CEA, CA50, and CA724 tended to have a more aggressive clinical stage and poorer tumor characteristics (*P* < *0.05*, Table 1).

### Nomograms for Predicting Survival Outcomes of CRC Patients

**Figure 3:**
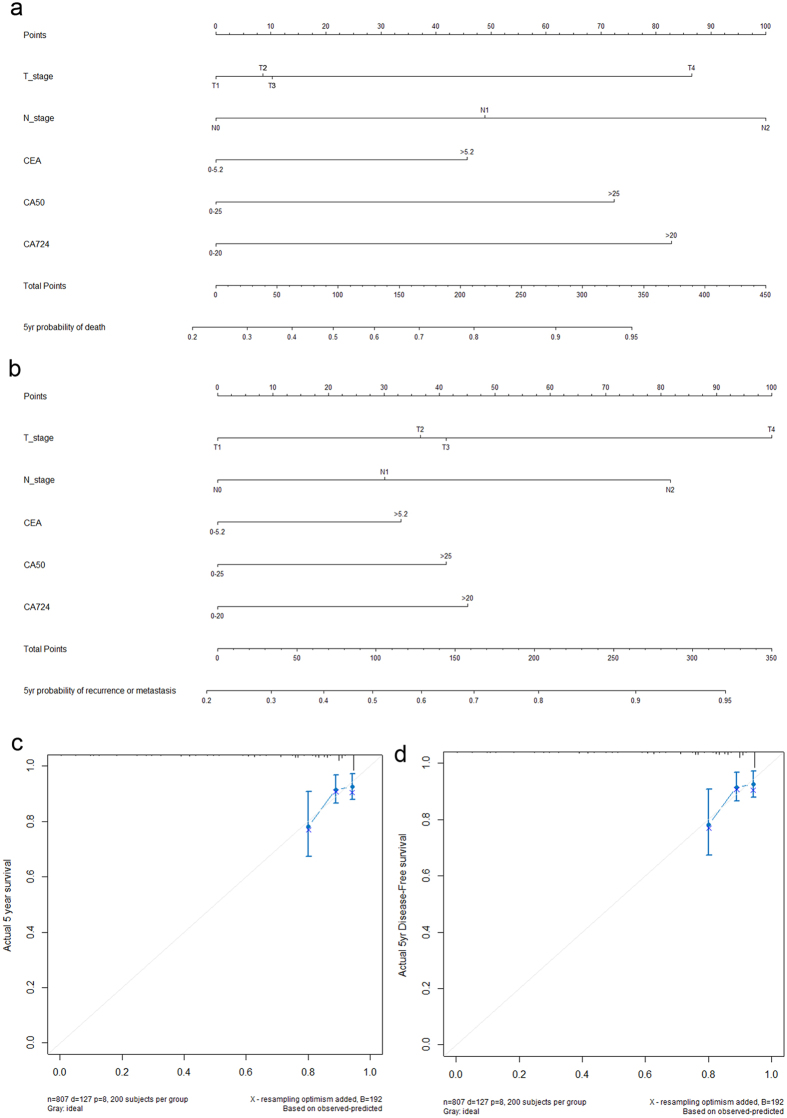
Nomograms deliver the results of prognostic models using clinicopathological features and serological tumor biomarkers to predict overall survival (**a**) and disease-free survival (**b**) of patients with colorectal cancer. Nomograms can be deciphered by adding the points assigned to each included variable shown at the top of the scale. The total points generate the predicted 5-year probability of death or recurrence/metastasis, as indicated on the lowest scale. The Harrell’s c-indexes for overall survival and disease-free survival prediction were 0.772 (95% CI: 0.724–0.820) and 0.715 (95% CI: 0.678–0.752), respectively. Calibration curves for 5-year overall survival (**c**) and 5-year disease-free survival (**d**) using nomograms with clinicopathological features and preoperative serological tumor markers are presented. The x-axis shows the predicted probability of survival and the y-axis shows actual survival. The reference line is 45°, indicating perfect calibration.

Based on the results of multivariable Cox regression analyses, two nomograms were established to predict OS and DFS (Fig. 3a,b). The c-indexes were 0.772 and 0.715 for predicting 5-year OS and DFS, respectively.

Figure 3c and d demonstrate the nomogram calibration curves. They were used to estimate how close the nomogram estimated risk was to the observed risk. Calibration was intuitively and typically assessed by reviewing the plot of predicted probabilities from the nomogram versus the actual probabilities. When examining the calibration plots in our study, we could see the points fell close to the reference line, and the width of CI was acceptable, which revealed good predictive ability.

### Subgroup Analysis of Tumor Markers According to CEA Status

CEA is the most commonly used biomarker in CRC. It is mainly employed during follow-up after radical surgery and may indicate recurrence and metastasis if elevated. However, the positive rate of CEA was only 38.8% in the present cohort. Therefore, subgroup analyses were conducted according to CEA status. In the CEA-high group, only CA724 was detected as an independent prognostic factor for OS [HR (hazard ratio) = 2.853, 95% CI (confidence interval): 1.769–4.603, *P* < 0.001] and DFS (HR = 2.059, 95% CI: 1.360–3.117, *P* = 0.001). In contrast, in the CEA-normal group, tumor biomarker CA50 was shown to be an independent risk factor for both OS (HR = 3.052, 95% CI: 1.105–8.430, *P* = 0.031) and DFS (HR = 2.416, 95% CI: 1.027–5.686, *P* = 0.043, Tables 3 and 4).

## Discussion

Distant metastases and local recurrences remain main concerns in CRC patients after surgical resection^8^,^9^. Identification of factors that are significantly associated with decreased survival would help the selection of patients at high risk of recurrences and metastases. Although the AJCC staging system is valuable for predicting prognosis and guiding treatments for patients with CRC, survival outcomes may be quite different even for patients at the same stage. Imaging techniques, which help estimate the therapeutic effect and constitute follow-up, are limited by cost and clinical experience, making them prone to miss small recurrences or metastases^10^. Thus, to optimize individualized disease management, it would be desirable to establish simple and cost-effective tools. Tumor biomarkers are substances expressed and released during tumorigenesis and progression and may indicate the presence of a new growth that implies the relative tumor burden and aggressive biology^10^,^11^. Because of its convenience and cost-effectiveness, tumor biomarkers are promising for guiding therapy schedules and monitoring recurrences and metastases.

Due to its simplicity and graphical representation, the nomogram has been widely adopted as a practical model for predicting prognosis. Each variable is assigned different points according to its weight, which is shown at the top of the scale. The total points of all included variables generate a numerical prediction of the 5-year probability of death or recurrence/metastasis for a patient, which is indicated on the lowest scale. The predictive accuracy of nomograms has been validated as favorable compared with the traditional TNM staging system for many malignancies. Nomograms can generate an individualized prediction of survival for patients, making it a practical tool for clinicians to identify patients at high risk for intensive follow-up.

The nomogram is a simple and visual statistical prediction model that produces a numerical probability of a survival event. By adding the points assigned to each factor, a predicted 5-year probability of death and recurrence or metastasis for a patient can be calculated and shown in the lowest scale^12^. In many malignancies, nomograms have been validated to have comparable ability to predict prognosis to the AJCC TNM staging system, thus making it an alternative or even a new standard^13^,^14^. The ability of nomograms to generate individualized predictions enables doctors to identify and stratify high-risk patients for intensive follow-up.

In the present study, we performed a retrospective study in patients with CRC who had all six tumor biomarkers examined preoperatively and found that CEA, CA50, and CA724 were independently associated with DFS and OS in patients with CRC after surgical resection. Then, we developed nomograms including tumor biomarker predictors and found that the nomograms could improve the prognosis prediction of CRC patients. Elevated serum CEA, CA50, and CA724 were associated with pathological stage and unfavorable clinicopathological characteristics such as venous invasion, tumor length, and tumor differentiation. Another finding of the present study the different prognostic models concerning tumor markers in CEA-high and CEA-normal CRC.

CA50 is a glycolipid antigen that plays an important role in cell growth and differentiation, indicating that tumor cells expressing this antigen may possess increased proliferative activity^15^. CA50 can also be observed in many cancers, especially in gastrointestinal malignancies^15^,^16^. Preoperative serum CA50 is of prognostic value for gastric cancer patients but is not an independent postoperative prognostic factor^16^. Although CA50 has been studied in CRC^6^,^15^, its prognostic value has not been clarified. Our study showed that CA50 was an independent prognostic factor for patients with CRC after radical resection. Subgroup analyses indicated that CA50 was the only tumor biomarker that was significantly correlated with long-term survival in CEA-normal CRC patients.

CA724 is a glycoprotein, with higher levels in gastric^17^,^18^, colorectal^19^, and breast^20^ cancer. Zhu *et al*. showed that there were statistically significant differences in CA724 levels between patients with and without distant metastases^19^. A meta-analysis demonstrated that CA724 was the most sensitive serum tumor biomarker for the detection of gastric cancer in the Chinese population^18^. However, in a small sample of breast cancer patients, there was no significant difference in CA724 levels in nipple discharge between patients with breast cancer and those with benign lesions^20^. Our study demonstrated that CA724 was correlated with advanced tumor stage and poor characteristics. CA724 was an independent predictor of CRC and may be specific for the CEA-high subgroup.

Tumor-related indicators in all individuals cannot be detected systematically and comprehensively due to limited economics and techniques^7^,^21^. Therefore, the joint detection of specific tumor biomarkers is of great significance^22^. Our study indicated that the expression of CEA, CA50, and CA724 has an important application for monitoring recurrence and metastasis. Nomograms based on CEA, CA50, and CA724 and other clinicopathological factors may be useful for predicting prognosis.

## Methods

### Study Population

Patients with histologically confirmed colorectal adenocarcinoma who received radical resection at Fudan University Shanghai Cancer Center (FUSCC) between January 1, 2007 and December 30, 2012 were included in the study. The database of FUSCC was built prospectively, and all information of CRC patients was recorded since January 2006^13^,^23^,^24^. The inclusion criteria were as follows: 1) patients were aged 18 years or older; 2) patients underwent radical resection; 3) patients were not treated with neoadjuvant chemotherapy or radiation; 4) patients had no other malignant tumors; and 5) CEA, CA19-9, CA242, CA72-4, CA50 and CA125 were tested before surgery. Because the proportion of patients with high AFP was very low, AFP was not included in the present study. Finally, 807 eligible patients were identified in this study.

Preoperative blood samples were drawn in the morning from all patients. Plasma was separated from the blood cells by centrifuging the blood sample at 1000 g for 10 minutes. Magnetic particle enzyme immunoassay using the UniCel DxI 800 Access immunoassay system (Beckman Coulter Inc., Fullerton, CA, USA) was performed to detect CEA, CA125, CA19-9, CA50, CA242, and CA72-4. According to the manufacturer’s instructions, 5.2 ng/mL, 35 U/mL, 27 U/mL, 25 U/mL, 20 U/mL, and 6.90 U/mL were chosen as cut-off values for serum CEA, CA125, CA19-9, CA50, CA242 and CA72-4, respectively.

Clinicopathological variables, such as age, sex, grade, histologic type, T and N stage, treatment type, regional lymph node harvest, and number of positive regional lymph nodes were retrieved from the FUSCC database. All patients were restaged according to the American Joint Committee on Cancer (AJCC) TNM Staging Classification for CRC (Seventh Edition, 2010). The primary study endpoints were overall survival (OS), which was calculated from the date of diagnosis to the date of death from any cause, and disease-free survival (DFS), which was defined as the time from diagnosis to the first recurrence. Patients who were alive at the last follow-up were censored for analyses.

### Ethics statement

This retrospective study was approved by the FUSCC Ethical Committee and Institutional Review Board. All methods were carried out in accordance with the relevant guidelines. All patients identified gave written informed consent for participation.

### Statistical Analyses

The relationship between clinicopathological features and tumor markers was tested by cross-tab analysis. The Kaplan-Meier estimator was used to calculate the 5-year overall survival (OS) and disease-free survival (DFS), with the difference between variables compared by log-rank tests. All prognostic predictors that were significantly associated with OS and DFS in univariate analysis were included in multivariate Cox proportional hazards analysis. *P* < *0.05* was considered statistically significant. Confidence intervals (CIs) at the 95% confidence level were used in this study.

Nomograms for prognostic parameters that were independently associated with OS and DFS were developed using R 3.1.2 software (Institute for Statistics and Mathematics, Vienna, Austria). Model performance for predicting survival probability was evaluated by Harrell’s concordance index (c-index). All statistical analyses were performed using SPSS 22.0 (SPSS Inc., Chicago, IL, USA).

## Additional Information

**How to cite this article**: Li, Q. *et al*. Nomograms for predicting prognostic value of serological tumor biomarkers in colorectal cancer patients after radical resection.. *Sci. Rep.* **7**, 46345; doi: 10.1038/srep46345 (2017).

**Publisher's note:** Springer Nature remains neutral with regard to jurisdictional claims in published maps and institutional affiliations.

## Figures and Tables

**Table 1:** Comparison of baseline clinicopathological characteristics based on CEA, CA50, and CA724.

	Cases	CEA (ng/mL)		*P*	CA50 (IU/mL)		*P*	CA724 (U/mL)		*P*
	No. (%)	≤5.20	>5.20		≤25	>25		≤20	>20	
Age (years)				0.002			0.379			0.130
≤60	427(52.9)	283(57.3)	144(46.0)		396(53.4)	31(47.7)		357(54.2)	70(47.3)	
>60	380(47.1)	211(42.7)	169(54.0)		346(46.6)	34(52.3)		302(45.8)	78(52.7)	
Gender				0.017			0.926			0.362
Male	215(26.6)	117(23.7)	98(31.3)		198(26.7)	17(26.2)		180(27.3)	35(23.6)	
Female	592(76.7)	377(42.6)	215(68.7)		544(73.3)	48(73.8)		479(72.7)	113(76.4)	
Tumor location				0.035			0.119			0.300
Colon	324(40.1)	184(37.2)	140(44.7)		292(39.4)	32(49.2)		259(39.3)	65(43.9)	
Rectum	483(59.9)	310(62.8)	173(55.3)		450(60.6)	33(50.8)		400(60.7)	83(56.1)	
Tumor length (cm)				0.001			0.025			<0.001
≤3 cm	276(35.4)	190(38.5)	86(27.5)		262(35.3)	14(21.5)		251(38.1)	25(16.9)	
>3 cm	531(63.2)	304(61.5)	227(72.5)		480(64.7)	51(78.5)		408(61.9)	123(83.1)	
T stage				<0.001			<0.001			0.004
T1	64(7.9)	59(11.9)	5(1.6)		63(8.5)	1(1.5)		60(9.1)	4(2.7)	
T2	146(18.1)	118(23.9)	28(8.9)		145(19.5)	1(1.5)		128(19.4)	18(12.2)	
T3	392(31.6)	226(45.7)	166(53.0)		361(48.7)	31(47.7)		313(47.5)	79(53.4)	
T4	205(25.4)	91(18.4)	114(36.4)		173(23.3)	32(49.2)		158(24.0)	47(31.8)	
N stage				<0.001			<0.001			0.002
N0	468(58.0)	326(66.0)	142(45.4)		447(60.2)	21(32.3)		396(60.1)	72(48.6)	
N1	202(25.0)	109(22.1)	93(29.7)		182(24.5)	20(30.8)		165(25.0)	37(25.0)	
N2	137(17.0)	59(11.9)	78(24.9)		113(15.2)	24(36.9)		98(14.9)	39(26.4)	
Differentiation				0.017			0.001			<0.001
Well/moderate	575(71.3)	353(71.5)	222(70.9)		540(72.8)	35(53.8)		499(75.7)	76(51.4)	
Poor	191(23.7)	108(21.9)	83(26.5)		163(22.0)	28(43.1)		128(19.4)	63(42.6)	
Unknown	41(5.1)	33(6.7)	8(2.6)		39(5.3)	2(3.1)		32(4.9)	9(6.1)	
Venous invasion				<0.001			<0.001			0.001
Negative	617(76.5)	404 (81.8)	213(68.1)		583(78.6)	34(52.3)		519(78.8)	98(66.2)	
Positive	190(23.5)	90(18.2)	100(31.9)		159(21.4)	31(47.7)		140(21.2)	50(33.8)	
Perineural invasion				<0.001			<0.001			0.071
Negative	1308(24.5)	968(26.4)	340(20.4)		620(83.6)	43(66.2)		549(83.3)	84(56.8)	
Positive	4028(75.5)	2700(73.6)	1328(79.6)		122(16.4)	22(33.8)		110(16.7)	64(43.2)	

**Table 2:** Univariate and multivariate survival analyses of OS and DFS in CRC patients of FUSCC.

	OS				DFS			
	Univariate analysis	*P*	Multivariate analysis	*P*	Univariate analysis	*P*	Multivariate analysis	*P*
	HR (95% CI)		HR (95% CI)		HR (95% CI)		HR (95% CI)	
Age (years)		0.071				0.292		
≤60	1.000				1.000			
>60	1.380 (0.973–1.957)				1.154 (0.884–1.508)			
Gender		0.251				0.524		
Male	1.000				1.000			
Female	1.289 (0.835–1.987)				0.907 (0.672–1.225)			
Tumor location		0.071				0.032		0.414
Colon	1.000				1.000		1.000	
Rectum	0.725 (0.512–1.027)				0.745 (0.570–0.974)		1.165 (0.807–1.682)	
Tumor length (cm)		0.002		0.250		0.012		0.952
≤3 cm	1.000		1.000		1.000		1.000	
>3 cm	1.918 (1.260–2.917)		1.296 (0.833–2.018)		1.468 (1.086–1.984)		1.010 (0.732–1.394)	
T stage		<0.001		<0.001		<0.001		<0.001
T1	1.000		1.000		1.000		1.000	
T2	1.445 (0.398–5.251)		1.046 (0.284–3.847)		1.913 (0.721–5.073)		1.591 (0.593–4.268)	
T3	2.812 (0.876–9.023)		0.930 (0.274–3.155)		3.344 (1.360–8.222)		1.655 (0.645–4.245)	
T4	7.995 (2.513–25.442)		2.280 (0.674–7.714)		7.685 (3.125–18.898)		3.773 (1.426–9.984)	
N stage		<0.001		<0.001		<0.001		<0.001
N0	1.000		1.000		1.000		1.000	
N1	2.464 (1.581–3.841)		1.794 (1.104–2.915)		1.965 (1.366–2.826)		1.309 (0.905–1.892)	
N2	5.149 (3.379–7.846)		2.946 (1.725–5.031)		4.831 (3.456–6.754)		2.266 (1.497–3.428)	
Differentiation		0.011		0.641		<0.001		0.962
Well/Moderate	1.000		1.000		1.000		1.000	
Poor	1.778 (1.221–2.589)		0.864 (0.572–1.306)		1.674 (1.253–2.236)		1.029 (0.753–1.407)	
Unknown	1.193 (0.550–2.587)		1.217 (0.550–2.694)		0.892 (0.455–1.752)		0.940 (0.473–1.869)	
Venous invasion		<0.001		0.589		<0.001		0.222
Negative	1.000		1.000		1.000		1.000	
Positive	3.312 (2.336–4.697)		1.133 (0.720–1.783)		2.841 (2.163–3.730)		1.249 (0.875–1.783)	
		<0.001		0.277		<0.001		0.058
Negative	1.000		1.000		1.000		1.000	
Positive	2.488 (1.722–3.594)		1.256 (0.833–1.892)		2.374 (1.779–3.169)		1.361 (0.990–1.870)	
CEA (ng/mL)		<0.001		**0.034**		<0.001		**0.017**
Normal (0–5.2)	1.000		1.000		1.000		1.000	
High (>5.2)	3.308 (2.294–4.768)		1.565 (1.035–2.368)		2.448 (1.868–3.209)		1.456 (1.071–1.980)	
CA199 (U/mL)		<0.001		0.458		<0.001		0.399
Normal (0–27)	1.000		1.000		1.000		1.000	
High (>27)	3.241 (2.287–4.593)		1.220 (0.721–2.065)		1.943 (1.466–2.575)		0.838 (0.556–1.263)	
CA50 (IU/mL)		<0.001		**0.010**		<0.001		**0.020**
Normal (0–25)	1.000		1.000		1.000		1.000	
High (>25)	5.288 (3.533–7.914)		1.905 (1.168–3.106)		3.322 (2.326–4.746)		1.647 (1.080–2.510)	
CA125 (U/mL)		<0.001		0.144		<0.001		0.212
Normal (0–35)	1.000		1.000		1.000		1.000	
High (>35)	3.410 (2.115–5.500)		1.491 (0.873–2.549)		2.329 (1.523–3.563)		1.337 (0.847–2.112)	
CA242		<0.001		0.683		<0.001		0.397
Normal (0–25)	1.000		1.000		1.000		1.000	
High (>35)	2.746 (1.938–3.892)		1.109 (0.676–1.819)		1.988 (1.511–2.615)		1.177 (0.807–1.718)	
CA724 (U/mL)		<0.001		<**0.001**		<0.001		**0.001**
Normal (0–20)	1.000		1.000		1.000		1.000	
High (>20)	3.467 (2.429–4.947)		2.515 (1.688–3.748)		2.330 (1.713–3.169)		1.723 (1.246–2.381)	

**Table 3:** Subgroup analyses of tumor antigens on OS and DFS in CEA-normal CRC patients.

	OS				DFS			
	Univariate analysis	*P*	Multivariate analysis	*P*	Univariate analysis	*P*	Multivariate analysis	*P*
	HR (95% CI)		HR (95% CI)		HR (95% CI)		HR (95% CI)	
Age (years)		0.725				0.776		
≤60	1.000				1.000			
>60	1.113 (0.615–2.014)				0.942 (0.622–1.426)			
Gender		0.730				0.148		
Male	1.000				1.000			
Female	1.138 (0.546–2.373)				0.714 (0.452–1.127)			
Tumor location		0.721				0.846		
Colon	1.000				1.000			
Rectum	1.120 (0.601–2.090)				1.043 (0.681–1.597)			
Tumor length (cm)		0.006		0.099		0.028		0.495
≤3 cm	1.000		1.000		1.000		1.000	
>3 cm	2.926 (1.360–6.295)		2.007 (0.877–4.594)		1.663 (1.056–2.618)		1.185 (0.728–1.927)	
T stage		<0.001		0.035		<0.001		0.016
T1	1.000		1.000		1.000		1.000	
T2	1.482 (0.299–7.344)		1.021 (0.198–5.258)		1.786 (0.588–5.428)		1.599 (0.518–4.930)	
T3	2.249 (0.520–9.737)		0.975 (0.204–4.649)		3.076 (1.105–8.563)		2.010 (0.684–5.907)	
T4	7.002 (1.631–30.067)		2.594 (0.523–12.881)		5.981 (2.105–16.991)		3.757 (1.222–11.555)	
N stage		<0.001		0.426		<0.001		0.109
N0	1.000		1.000		1.000		1.000	
N1	1.292 (0.592–2.820)		0.864 (0.367–2.035)		1.668 (1.014–2.743)		1.262 (0.732–2.175)	
N2	4.100 (2.083–8.068)		1.566(0.613–3.996)		3.677 (2.234–6.053)		1.964 (1.046–3.689)	
Differentiation		0.157				0.227		
Well/Moderate	1.000				1.000			
Poor	1.848 (0.965–3.542)				1.458 (0.921–2.309)			
Unknown	1.634 (0.570–4.684)				0.844 (0.339–2.101)			
Venous invasion		0.001		0.052		0.001		0.178
Negative	1.000		1.000		1.000		1.000	
Positive	2.774 (1.500–5.127)		2.195 (0.993–4.855)		2.169 (1.384–3.398)		1.455 (0.843–2.510)	
Perineural invasion		0.461				0.025		0.468
Negative	1.000				1.000		1.000	
Positive	1.355 (0.604–3.040)				1.800 (1.075–3.012)		1.226 (0.708–2.122)	
CA199 (U/mL)		0.001		0.194		0.049		0.449
Normal (0–27)	1.000		1.000		1.000		1.000	
High (>27)	3.079 (1.611–5.886)		1.783 (0.745–4.270)		1.699 (1.003–2.877)		0.760 (0.374–1.545)	
CA50 (IU/mL)		<0.001		**0.031**		0.001		0.043
Normal (0–25)	1.000		1.000		1.000		1.000	
High (>25)	7.183 (3.024–17.060)		3.052 (1.105–8.430)		3.869 (1.787–8.378)		2.416 (1.027–5.686)	
CA125 (U/mL)		0.292				0.799		
Normal (0–35)	1.000				1.000			
High (>35)	1.878 (0.581–6.065)				1.139 (0.418–3.103)			
CA242		0.024		0.245		0.010		0.139
Normal (0–25)	1.000		1.000		1.000		1.000	
High (>35)	2.146 (1.105–4.168)		0.972 (0.406–2.330)		1.885 (1.167–3.046)		1.614 (0.856–3.044)	
CA724 (U/mL)		0.012		0.093		0.274		
Normal (0–20)	1.000		1.000		1.000			
High (>20)	2.392 (1.209–4.734)		1.828 (0.904–3.696)		1.362 (0.783–2.369)			

**Table 4:** Subgroup analyses of tumor antigens on OS and DFS in CEA-high CRC patients.

	OS				DFS			
	Univariate analysis	*P*	Multivariate analysis	*P*	Univariate analysis	*P*	Multivariate analysis	*P*
	HR (95% CI)		HR (95% CI)		HR (95% CI)		HR (95% CI)	
Age (years)		0.235				0.452		
≤60	1.000				1.000			
>60	1.303 (0.841–2.019)				1.147 (0.803–1.639)			
Gender		0.060				0.248		
Male	1.000				1.000			
Female	1.670 (0.978–2.851)				1.265 (0.849–1.887)			
Tumor location		0.077				0.012		0.405
Colon	1.000				1.000		1.000	
Rectum	0.676 (0.438–1.043)				0.636 (0.446–0.907)		0.807 (0.486–1.338)	
Tumor length (cm)		0.503				0.747		
≤3 cm	1.000				1.000			
>3 cm	1.188 (0.718–1.965)				1.069 (0.714–1.599)			
T stage		0.001		0.004		<0.001		0.063
T1	1.000		1.000		1.000		1.000	
T2	0.670 (0.075–6.001)		1.063 (0.113–9.978)		1.360 (0.167–11.056)		1.371 (0.166–11.314)	
T3	0.892 (0.122–6.534)		0.553 (0.072–4.263)		1.570 (0.217–11.365)		0.871 (0.117–6.479)	
T4	2.066 (0.284–15.004)		1.320 (0.172–10.111)		3.598 (0.499–25.941)		1.727 (0.220–13.525)	
N stage		<0.001		0.001		<0.001		0.002
N0	1.000		1.000		1.000		1.000	
N1	2.954 (1.645–5.303)		2.497 (1.334–4.675)		1.765 (1.122–2.777)		1.360 (0.822–2.250)	
N2	4.550 (2.573–8.048)		1.566(1.817–7.051)		3.150 (2.049–4.842)		2.566 (1.482–4.444)	
Differentiation		0.118				0.008		0.878
Well/Moderate	1.000				1.000		1.000	
Poor	1.625 (1.025–2.575)				1.797 (1.235–2.615)		1.095 (0.730–1.643)	
Unknown	1.273 (0.395–4.101)				1.555 (0.569–4.250)		0.914 (0.321–2.599)	
Venous invasion		<0.001		<0.001		<0.001		0.484
Negative	1.000		1.000		1.000		1.000	
Positive	2.940 (1.906–4.534)		3.013 (2.090–4.344)		2.772 (1.943–3.953)		1.185 (0.736–1.907)	
Perineural invasion		<0.001		<0.001		<0.001		0.038
Negative	1.000		1.000		1.000		1.000	
Positive	2.285 (1.480–3.528)		2.019 (1.388–2.936)		2.142 (1.492–3.076)		1.530 (1.023–2.289)	
CA199 (U/mL)		0.001		0.775		0.084		
Normal (0–27)	1.000		1.000		1.000			
High (>27)	2.102 (1.361–3.247)		1.094 (0.592–2.019)		1.367 (0.959–1.949)			
CA50 (IU/mL)		<0.001		0.078		<0.001		0.118
Normal (0–25)	1.000		1.000		1.000		1.000	
High (>25)	3.063 (1.919–4.887)		1.673 (0.944–2.963)		2.182 (1.441–3.303)		1.429 (0.913–2.236)	
CA125 (U/mL)		0.292		0.122		<0.001		0.158
Normal (0–35)	1.000		1.000		1.000		1.000	
High (>35)	3.085 (1.808–5.266)		1.639 (0.877–3.065)		2.367 (1.463–3.828)		1.466 (0.862–2.492)	
CA242		0.003		0.538		0.095		
Normal (0–25)	1.000		1.000		1.000			
High (>35)	1.964 (1.268–3.042)		1.201 (0.671–2.149)		1.352 (0.949–1.925)			
CA724 (U/mL)		<0.001		<0.001		<0.001		0.001
Normal (0–20)	1.000		1.000		1.000		1.000	
High (>20)	3.096 (2.009–4.772)		2.853 (1.769–4.603)		2.166 (1.506–3.114)		2.059 (1.360–3.117)	
